# Infection with different human influenza A subtypes affects the period of susceptibility to secondary bacterial infections in ferrets

**DOI:** 10.1093/femsmc/xtac011

**Published:** 2022-04-29

**Authors:** Edin J Mifsud, Rubaiyea Farrukee, Aeron C Hurt, Patrick C Reading, Ian G Barr

**Affiliations:** WHO Collaborating Centre for Reference and Research on Influenza, Peter Doherty Institute for Infection and Immunity, Victoria 3010, Australia; Department of Microbiology and Immunology, Peter Doherty Institute for Infection and Immunity, University of Melbourne, Victoria 3010, Australia; Department of Microbiology and Immunology, Peter Doherty Institute for Infection and Immunity, University of Melbourne, Victoria 3010, Australia; WHO Collaborating Centre for Reference and Research on Influenza, Peter Doherty Institute for Infection and Immunity, Victoria 3010, Australia; WHO Collaborating Centre for Reference and Research on Influenza, Peter Doherty Institute for Infection and Immunity, Victoria 3010, Australia; Department of Microbiology and Immunology, Peter Doherty Institute for Infection and Immunity, University of Melbourne, Victoria 3010, Australia; WHO Collaborating Centre for Reference and Research on Influenza, Peter Doherty Institute for Infection and Immunity, Victoria 3010, Australia; Department of Microbiology and Immunology, Peter Doherty Institute for Infection and Immunity, University of Melbourne, Victoria 3010, Australia

**Keywords:** viral bacterial coinfections, ferrets, *Streptococcus pneumoniae*, community acquired pneumonia, Influenza A virus, septicemia

## Abstract

It is well-established that influenza virus infections predispose individuals to secondary bacterial infections (SBIs), which may result in a range of clinical outcomes from relatively mild (e.g. sinusitis and otitis media) to severe (e.g. pneumonia and septicaemia). The most common bacterial pathogen associated with SBI following influenza virus infections is *Streptococcus pneumoniae*(SPN). Of circulating human seasonal influenza viruses, influenza A viruses (IAV) of both the A(H1N1)pdm09 and A(H3N2) subtypes are associated with severe disease but have differing hospitalisation and complication rates. To study the interplay of these two IAV subtypes with SBI, we used a ferret model of influenza infection followed by secondary challenge with a clinical strain of SPN to determine the severity and the period of susceptibility for SBI. Ferrets challenged with SPN 5 days after infection with A(H3N2) or A(H1N1)pdm09 viruses developed severe disease that required euthanasia. When the time between viral infection and bacterial challenge was extended, A/H1N1pdm09-infected animals remained susceptible to SBI- for up to 10 days after the viral infection. For A(H3N2)- but not A(H1N1)pdm09-infected ferrets, susceptibility to SBI-associated disease could be extended out to 16 days postviral infection. While caution should be taken when extrapolating animal models to human infections, the differences between A(H3N2) and A(H1N1)pdm09 strains in duration of susceptibility to SBI observed in the ferret model, may provide some insight regarding the higher rates of SBI-associated disease associated with some strains of A(H3N2) viruses in humans.

## Teaser

Secondary bacterial infections are a serious complication following influenza and the window of susceptibility to subsequent bacterial infections differs following infection with either H1N1 or H3N2 influenza viruses.

## Introduction

Currently, influenza A viruses (IAV) and influenza B viruses (IBV) cocirculate and cause significant disease in humans (Bailey et al. 2018). IAVs currently circulating in humans can be further divided into the A(H3N2) and A(H1N1)pdm09 subtypes, while IBV are classified into the B/Yamagata- and B/Victoria-lineages. Virus replication in the lower respiratory tract (LRT) can result in primary viral pneumonia, often requiring hospitalisation (Morens et al. 2008). In regard to IAV infections, epidemiological studies have demonstrated higher hospitalisation rates and exacerbated disease severity associated with A(H3N2) compared to A(H1N1) or A(H1N1)pdm09 infections (Krammer et al. 2018, Rello and Pop-Vicas 2009, Hayward et al. 2014, Kwok et al. 2017, Burrell et al. 2018). This was most evident during the 2017 influenza season, where the predominant circulating strain was A(H3N2), and hospitalisations and ICU admissions were greater than those observed during the peak of the A(H1N1)pdm09 pandemic in 2009 (Burrell et al. 2018). Furthermore, admissions associated with influenza and sepsis was more common during the 2017 A(H3N2) season (53.4%) when compared to the A(H1N1)pdm09 pandemic in 2009 (36.8%; Burrell et al. 2018).

The most severe complication associated with influenza infections is the development of secondary bacterial infections (SBIs) caused by a range of bacteria including *Streptococcus pneumoniae* (SPN), *Haemophilus influenzae*, *Staphylococcus aureus*, and *Streptococcus pyogenes* (Sheykhsaran et al. 2019*)*. In both adult and paediatric populations, between 30% and 50% of hospitalised cases with a laboratory-confirmed influenza diagnosis, also presented with a bacterial infection detected in the nasopharynx (Diaz et al. 2016, Self et al. 2016, Voiriot et al. 2016, Cawcutt and Kalil 2017). Observational studies examining the outcomes of bacterial coinfection following influenza in adults were associated with a 4-fold increase in mortality (Rozencwajg et al. 2018), while another study assessing clinical characteristics of dual infections in children with influenza-related acute respiratory failure found that mortality rates were increased 9-fold (Randolph et al. 2019) iin children with methicillin-resistant *S. aureus* SBI.

SPN has been the most common bacteria found to be associated with SBIs (Morens et al. 2008) and historically it has been linked to exacerbation of disease during influenza epidemics and pandemics, including the 1918 Spanish flu (Brundage 2006, Joseph et al. 2013). The mechanisms by which the host is rendered more susceptible to SBIs postinfluenza has been the subject of numerous studies. One suggested mode has been *via* epithelial cell damage as a result of cytolytic viral infection or *via* host-induced inflammation, which can result in upregulation of the host receptor molecules that SPN utilise for attachment and colonisation (Håkansson et al. 1994, Peltola and McCullers 2004). Influenza virus infection has also been associated with suppressed function of airway macrophages, neutrophils, and dendritic cells (DC; Williams et al. 2004, Brundage 2006, Shahangian et al. 2009) by virus-induced reduction in TLR-activation (Williams et al. 2004), thereby promoting propagation and entry of SPN into sterile sites of the body. Moreover, influenza virus-induced immune cell suppression has been shown to last for prolonged periods of time (Williams et al. 2004, Didierlaurent et al. 2008). Despite clear evidence of influenza virus-induced alterations to the respiratory tract, including enhanced bacterial colonisation (Wadowsky et al. 1995, Wu et al. 2011, Wren et al. 2014), the duration of susceptibility to SBIs following influenza virus infections is unclear.

Ferrets represent a useful model to study SBI following influenza virus infections to bridge the gap between studies in mice and humans. Unlike mice, ferrets are outbred animals that show similar clinical signs following influenza infections to those observed in humans including raised body temperature, lethargy, and virus shedding from the URT for up to a week. Moreover, ferrets can also be infected with human influenza viruses without prior adaption [reviewed in Oh and Hurt (2016)]. Ferrets have also been utilised to examine some aspects of dual influenza and SBI, such as the development of otitis media, sinusitis, and/or pneumonia following intranasal infection with SPN after an initial influenza infection (Peltola et al. 2006, McCullers et al. 2010). These studies highlight the potential of the ferret model for further studies examining SBI-associated disease induced following influenza infections.

This study aimed to determine the duration of susceptibility to SBI-associated disease following SPN infection in the ferret model using two different IAV subtypes, namely [A(H1N1)pdm09 and A(H3N2)]. We hypothesised that the duration of susceptibility may differ between IAV subtypes, and that this might correlate with the different hospitalisation rates associated with infections by different subtypes as has been reported in human cases.

## Materials and methods

### Influenza viruses

Clinical samples containing influenza viruses A/Perth/265/2009 (H1N1)pdm09, and A/Sydney/1234/2019 (H3N2) used in the study were submitted to the WHO Collaborating Centre for Reference and Research on Influenza, Melbourne, Australia, as part of the WHO Global Influenza Surveillance and Response System (GISRS). A/Perth/265/2009 was propagated in Madin-Darby canine kidney (MDCK) cells obtained from the American Type Culture Collection (CCL-34 Manassas, VA, USA). A/Sydney/1234/2019 was propagated in MDCK-SIAT1 cells (kindly provided by Professor Hans-Dieter Klenk, University of Marburg, Marburg, Germany). Both cell lines were passaged in Dulbecco's modified Eagle's medium/HAM's F12 Coon's medium (SAFC Biosciences) supplemented with 10% (v/v) fetal calf serum (FCS), 2 mM l-glutamine, nonessential amino acids, 0.075% (v/v) sodium bicarbonate, 10 mM HEPES, 50 U/ml penicillin,  50 μg/ml streptomycin (SAFC Biosciences), and 20 μg/ml amphotericin B (Fungizone; Bristol-Myers Squibb Company). The passaging medium for MDCK-SIAT1 cells was supplemented with 1 mg/ml G418 sulphate (Geneticin;  Gibco). Viral infection studies were performed in the absence of FCS and G418. Cells were maintained at 37°C before infection and at 35°C following infection.

### Bacteria

*Streptococcus pneumonia* isolate EF3030 (serotype 19F) was kindly provided by Associate Professor Catherine Satzke and Dr Salvatore Manna, and was prepared as previously described (Diavatopoulos et al. 2010).

### Animal ethics statement

Experiments using ferrets were conducted with the approval of The University of Melbourne Animal Ethics Committee (AEC number 1814559) in strict accordance with the Australian Government, National Health and Medical Research Council Australian code of practice for the care and use of animals for scientific purposes (eighth edition).

### Ferrets

Outbred 6-month-old adult male and female ferrets (Animalactic Animals and Animal Products Pty Ltd, Victoria, Australia) weighing more than 600 g were used in all experiments. Serum samples from ferrets were tested by hemagglutination inhibition assay against the reference strains A/California/7/2009 (H1N1)pdm09, A/Victoria/361/2011 (H3N2), B/Wisconsin/1/2010 (B/Yamagata-lineage), and B/Brisbane/60/2008 (B/Victoria-lineage) using turkey erythrocytes, to ensure seronegativity against currently circulating influenza subtypes and lineages. Throughout experiments, ferrets were housed individually in high efficiency particulate air-filtered cages with *ad libitum* food, water, and with added enrichment equipment.

Ferrets (4–10 per group) were anaesthetised [50:50 mix of Ketamine (100 mg/ml): Ilium Xylazil (Xylazine; 20 mg/ml)] prior to intranasal inoculation with 500 µl of 10^3^ TCID_50_ (50% of the tissue culture infectious dose) of A/Perth/265/2009 (A(H1N1)pdm09) or 10^5^ TCID_50_ of A/Sydney/1234/2019 or A/Udorn/307/1972 (A(H3N2)) viruses. A higher virus dose was used for A(H3N2) viruses compared to the A(H1N1)pdm09 virus, to ensure recoverable viral titres were obtained during nasal washing.

Animals were monitored daily following infection for any signs of respiratory illness. At 5 , 10, or 16 days following influenza infection, animals were inoculated intranasally with 10^3^ colony-forming units/ml (CFU) of SPN in a small volume (50 µl/dose) to ensure that bacteria were deposited into the upper respiratory tract (URT) only. Animals were monitored twice daily following bacterial infection for any signs of illness that necessitated euthanasia. Animals were euthanised if they were inactive, showed signs of laboured breathing, severe dehydration, or significant loss of body condition (> 15% weight loss) or had a temperature more than 2° above their original body temperature.

### Sample collection

Nasal washes were collected daily from mildly sedated ferrets (intramuscular injection of Xylazine at 5 mg/kg) following influenza virus and/or SPN challenge by instilling 1 ml of sterile PBS into one nostril and allowing the liquid to flow out of the other nostril into a collection tube. Aliquots of nasal washes were stored at −80°C prior to determining viral titres. Ferrets were sacrificed at various time points postbacterial infection by intramuscular injection of anaesthesia [50:50 mix of Ketamine (100 mg/ml): Ilium Xylazil (Xylazine; 20 mg/ml)] followed by an overdose of pentobarbitone sodium (Lethabarb; 0.5 ml/kg). For LRT samples, all five lobes of the lung were collected, snap frozen, and immediately stored at −80°C for TCID_50_ and bacterial colony counts.

### Virological analysis

Each individual lung lobe was homogenised using the gentleMACS^TM^ dissociator (MACS Miltenyi Biotec, Australia) in 5 ml of PBS prior to determining infectious viral titres. Titres of infectious virus in the nasal washes and lung samples were quantified by a viral infectivity assay, TCID_50_ as described previously (Hurt et al. 2010) and endpoints were calculated as described by Reed and Muench (Monto and Maassab 1981).

### Bacteriological analysis

After separately homogenising each lung lobe in PBS, 100 µl was plated on horse blood agar (HBA) plates, then samples were further diluted 1/10 and 100ul was plated on HBA. URT samples were also serially diluted to 1/10, and 100 µl was used for bacterial colony enumeration. Plates were then incubated at 37°C, 5% CO_2_ for 18–24 hours, and the bacterial colonies were manually enumerated and represented as colony-forming units/ml.

### Statistical analysis

Unpaired Student's *t*-test with nonparametric Mann–Whitney correction was used to compare day-to-day differences (body temperature and body weight to mean baseline/day 0 value) and between the different groups (nasal wash cell count, virus titre). A *P*-value of < .05 was considered statistically significant. All statistical analysis was performed using GraphPad Prism Version 9.0.0.

## Results

### Ferrets remain susceptible to SPN for 10 days after infection with A/(H1N1)pdm09 influenza virus

To determine the duration of susceptibility to bacterial infections postinfluenza infection, animals were first infected with A/Perth/265/2009 ((A(H1N1)pdm09) and subsequently inoculated with SPN at various times postviral challenge. The 19F strain of SPN was used as it is known to colonise the URT of children and adults (Brueggemann et al. 2004) and to cause invasive lung disease (Sandgren et al. 2005), and has been used in other studies in animal models (Sharma-Chawla et al. 2016).

**Figure 1.**
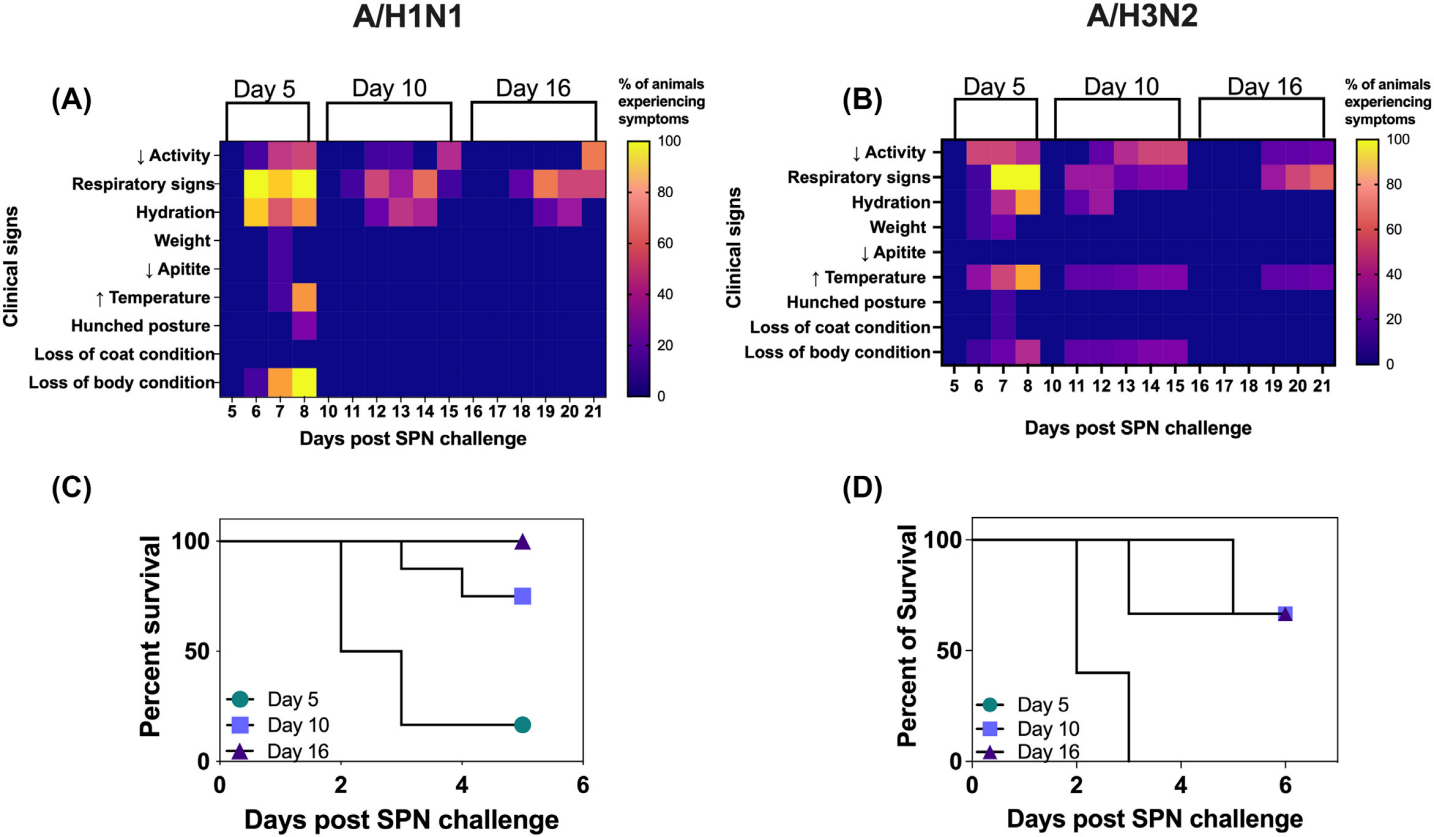
Clinical signs and survival in animals inoculated with SPN at various times following infection with A(H1N1)pdm09 strain A/Perth/265/2009 or A(H3N2) strain A/Sydney/1234/2019. Ferrets were infected *via* the intranasal route with either A/Perth/265/2009 (10^3^ TCID_50_) or A/Sydney/1234/2019 (10^5^ TCID_50_) in 500 µl of PBS. At 5 (circle), 10 (square), or 16 (triangle) days after viral infection, ferrets were inoculated with 10^3^ CFU of SPN in 50 µl of PBS. Animals were monitored daily for clinical signs. **(A)** and **(B)** Heat maps show the percentage of animals experiencing clinical signs. Amongst these are (i) reduced activity, (ii) respiratory signs, which include nasal discharge, laboured breathing, coughing, and sneezing, (iii) dehydration, (iv) loss of weight, (v) elevated temperatures, (vi) changes in posture, such as hunched disposition, and (vii) loss of coat or body condition. Survival following bacterial infection is shown in **(C)** and **(D)**. Symbols represent the percentage of surviving animals.

At day 5 after A(H1N1)pdm09 infection, inoculation with SPN did not result in weight loss or elevated body temperatures (Fig. 1A), but was associated with other signs of illness, including inactivity (50%, 3/6), dehydration (83%, 5/6), and respiratory symptoms (100%, 6/6) such as laboured breathing, nasal discharge, and coughing (Fig. 1A). In these experiments, 83% of animals (5/6) required euthanasia as early as 48 hours after challenge with SPN (Fig. 1C). Animals challenged with SPN 10 days after A(H1N1)pdm09 infection also did not show any changes in body temperature or weight (Fig. 1A and C), but some animals displayed respiratory symptoms (44%, 4/9) and dehydration (33%, 3/9) by day 3 post-SPN challenge. Moreover, 22% of animals (2/9) were euthanised at the humane endpoint for these experiments at day 3 post-SPN challenge (Fig. 1C). Conversely, animals challenged with SPN 16 days after A(H1N1)pdm09 infection displayed only mild symptoms, including dehydration (33%, 2/6) and some minor respiratory symptoms (50%, 3/6, Fig. 1A), and none required euthanasia prior to the experimental endpoint (day 6 post-SPN challenge). Animals that received SPN alone showed no signs of illness at days 5, 10, and 16 post-SPN challenge. Overall, these data suggest that for up to 10 days after A(H1N1)pdm09 infection, ferrets showed heightened susceptibility to subsequent challenge with SPN and that the severity of disease was reduced at longer intervals between A(H1N1pdm)09 infection and subsequent SPN challenge.

**Figure 2.**
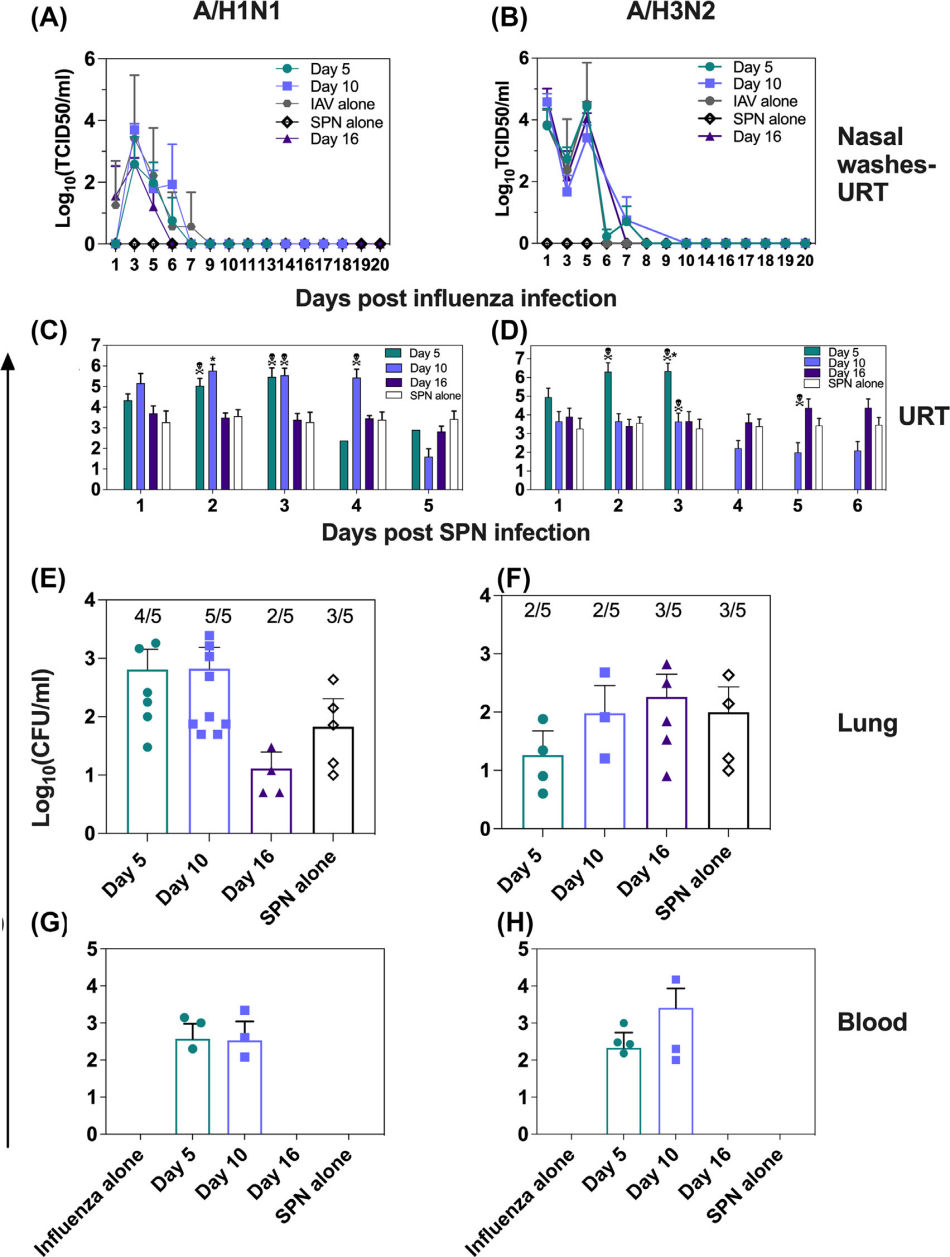
Viral and bacterial burden in animals challenged with SPN at various times following A/H1N1pdm09 and A/H3N2 infection. Ferrets were infected with A/Perth/265/2009 (10^3^ TCID_50_) or A/Sydney/1234/2019 (10^5^ TCID_50_) in 500 µl of PBS. **(A)** and **(B)** Influenza virus titres in the URT were enumerated using a TCID_50_ assay. Symbols represent the mean virus titre (± SD). **(C)** and **(D)** At 5, 10, or 16 days after viral infection, all animals except for IAV alone were challenged with 10^3^ CFU of SPN in 50 µl PBS. Note that a control group (SPN alone) was included in which animals were challenged with SPN in the absence of prior IAV infection. Following bacterial inoculation, nasal washes were collected daily. Bacterial titres in nasal wash samples were determined on HBA plates. **(E)**, **(F)**, **(G)**, and **(H)** Following euthanasia, lungs and blood were harvested and bacterial titres determined. Bars represent the mean bacterial titre (± SD) and symbols show titres in individual animals. The average number of lung lobes with detectable bacteria per group are shown above bars in (C) and (D). Skull and bones symbol (**) represents the time point where an animal was euthanised. Statistical significance was determined using a Mann–Whitney Student's *t*-test compared to SPN alone controls.

### Elevated bacterial burden in ferrets infected with A(H1N1)pdm09 prior to SPN challenge

Following A(H1N1)pdm09 infection, animals were monitored daily and nasal wash samples obtained every second day (Fig. 2A). Virus titres peaked at 3 days postinfection and were cleared from the URT by day 7. Based on these results, we concluded that only animals challenged with SPN 5 days after influenza infection had concurrent infections. No signs of viral ‘rebound’ (i.e. a subsequent increase in virus titres in the URT) were observed in these animals following SPN challenge. Nasal wash samples obtained daily from animals following SPN challenge 5 days after IAV infection showed peak bacterial load 2 days later and were significantly elevated (∼100–1000-fold CFU/ml) compared to uninfected animals challenged with SPN (Fig. 2C). The elevated bacterial load in this group 2–3 days after SPN challenge correlated with enhanced mortality. Bacterial titres recovered from the sole surviving animal in this group were comparable to the uninfected control group challenged with SPN alone.

At the time of euthanasia, the lungs and blood were harvested from animals to enumerate bacterial load. As the bacterial inoculum had been delivered in 50 µl to ensure that bacteria were deposited only in the URT, the presence of bacteria in the lungs or blood would indicate bacterial replication and spread. Extrapulmonary spread of bacteria is often associated with severe disease and is believed to be a major contributor to SBI-associated mortality (Iwashyna et al. 2012). Ferret lungs were harvested and bacterial titres determined in clarified homogenates prepared from each of the 5 lung lobes per animal. Bacteria could be detected in one or more lung lobes from 5/6 animals challenged with SPN 5 days after A(H1N1)pdm09 infection (Fig. 2E). On average, bacteria were recovered from four lung lobes per animal, suggestive of diffuse bacterial replication throughout the lungs. Septicaemia was evident in 3/6 animals in this group (Fig. 2F).

High bacteria loads were detected in the URT of all nine animals challenged with SPN 10 days after IAV infection, consistently ∼1000-fold higher at day 4 post-SPN challenge than in control (SPN alone) animals (Fig. 2C). However, by day 5 post-SPN challenge, bacterial loads in the URT were comparable to controls (SPN alone). Irrespective of high bacterial load, only two animals succumbed to infection in this group and SPN was detected in all lung lobes from 9/9 animals (Fig. 2E). Septicaemia was also evident in 3/9 animals (Fig. 2G), these animals were euthanised. Overall, animals challenged with SPN 10 days after A(H1N1)pdm09 infection showed reduced mortality compared to animals challenged with SPN 5 days after virus infection.

SPN challenge of animals 16 days after A(H1N1)pdm09 infection resulted in bacterial loads in nasal wash samples that were comparable to those in control animals (Fig. 2C). Bacterial loads in the lungs were also low and comparable to SPN controls (Fig. 2E) and none of these animals became septicaemic.

Together, these data suggest that SPN challenge following A(H1N1)pdm09 infection leads to exacerbated disease outcomes for up to 10 days after infection. However, exacerbated disease is not observed if animals are challenged with SPN 16 days after A(H1N1)pdm09 infection.

### Ferrets remain susceptible to SPN at least 16 days after infection with A(H3N2) influenza virus

**Figure 3.**
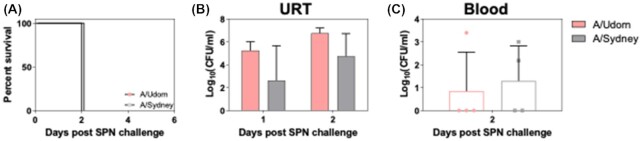
Bacterial burden in animals infected with different A(H3N2) IAV strains 5 days prior to SPN challenge. Ferrets were infected with either A/Udorn/307/1972 or A/Sydney/1234/2019 (10^5^ TCID_50_) in 500 µl PBS and monitored daily. A total of 5 days after viral infection, ferrets were inoculated with 10^3^ CFU of SPN in 50 µl of PBS. (A) Survival over time following bacterial inoculation. (B) Nasal wash samples were taken daily and the bacterial load determined on HBA plates. (C) Following euthanasia, blood was collected and the bacterial load determined. Bars represent the mean bacterial titre (± SD) and symbols show titres in individual animals.

Following intranasal infection, most contemporary A(H3N2) strains do not replicate in the LRT of ferrets (Cheng et al. 2012). To determine if replication of IAV in the LRT was required for severe disease following subsequent SPN challenge, ferrets were infected with A(H3N2) strains A/Udorn/307/1972 and A/Sydney/1234/2019 A(H3N2) to assess virus growth in the upper and lower airways. At day 5 postviral infection, we observed replication of A/Udorn/307/1972, but not A/Sydney/1234/2019, in ferret lungs (3.41 ± 1.45 vs. < 2 TCID_50_/ml (limit of detection), respectively). Based on these findings, we designed an experiment to examine the impact of SPN challenge following infection of ferrets with A(H3N2) strains that did or did not replicate in the LRT. Therefore, ferrets were infected with A/Udorn/307/1972 or A/Sydney/1234/2019 and, 5 days later, challenged with 10^3^ CFU/ml of SPN in 50 µl of PBS. Irrespective of the A(H3N2) strain used, all animals developed severe disease, necessitating euthanasia 2 days post-SPN challenge (Fig. 3A). Bacterial burden in the URT was comparable between groups, as was the incidence of septicaemia (Fig. 3B and C). Overall, these data indicated that IAV replication in the lungs was not a requirement for severe disease to occur following a subsequent SPN challenge. Given these findings, further experiments were performed using the contemporary A(H3N2) A/Sydney/1234/2019 A(H3N2) virus strain.

Next, ferrets were infected with 10^5^ TCID_50_ of A/Sydney/1234/2019 and subsequently inoculated with SPN at day 5, 10, or 16 post-IAV infection. Bacterial challenge 5 days postinfluenza infection was not associated with significant weight loss (Fig. 1B), but resulted in elevated body temperatures (80%, 8/10 animals), a decline in body condition (40%, 4/10), dehydration (80%, 8/10), and reduced activity (4/10, 40%), as well as severe respiratory symptoms (100%, 6/6; Fig. 1B). All animals were euthanised 72 hours post-SPN challenge (Fig. 1D). Animals challenged with SPN at day 10 postviral infection also resulted in some animals with elevated body temperature (33%, 2/6), decline in body condition (33%, 2/6), and respiratory signs (33%, 2/6). The two animals that presented with these signs were euthanised 72 hours after bacterial inoculation (Fig. 1D) and the other four animals without clinical signs survived until the end of the experiment at day 6 post-SPN challenge.

Of interest, some animals challenged with SPN 16 days after A(H3N2) infection also presented with several clinical signs, including respiratory signs (33%, 2/6) and elevated body temperatures (33%, 2/6). Animals that presented with signs of disease were euthanised 5 days after bacterial challenge (Fig. 1D). Overall, these data suggest that animals infected with either A(H1N1)pdm09 or A(H3N2) viruses exhibited distinct susceptibility to disease, with some A(H3N2)-infected ferrets remaining susceptible to SBI-induced disease for longer time periods following initial viral infection.

### Elevated bacterial burden in animals initially infected with A(H3N2) prior to SPN challenge

Following A/Sydney/1234/2019 A(H3N2) viral infection, animals were monitored daily, and nasal wash samples were obtained every second day. For all animals infected with the influenza virus, a biphasic peak in viral titres was observed in the URT, peaking at days 1 and 3 postviral infection (Fig. 2B). In A(H3N2)-infected animals, virus was cleared from the URT by day 7 days postinfection. Therefore, only animals challenged with SPN 5 days after A(H3N2) infection had concurrent infections and no signs of viral rebound were observed in these animals following SPN inoculation.

Following SPN challenge, nasal wash samples were obtained daily, and bacterial titres determined. Bacterial burden in animals challenged with SPN 5 days after A(H3N2) infection showed a similar pattern to animals infected with A(H1N1)pdm09 5 days prior to SPN challenge (Fig. 2C and D). Bacterial loads peaked at day 2 and remained elevated at day 3 post-SPN challenge, and were significantly elevated at day 3 compared to SPN control animals (Fig. 2D). The elevated bacterial load in this group correlated with enhanced mortality and all animals were euthanised by day 2 post-SPN challenge. In these ferrets, bacteria were detected in one or more lung lobes in 4/9 animals at the time of euthanasia (Fig. 2F). On average, bacteria were recovered from two lung lobes per animal (Fig. 2F), suggesting less bacterial replication in the lungs of animals infected previously with A(H3N2) compared to A(H1N1)pdm09 (4/9 (44%) vs. 5/6 (83%) animals, respectively). Again, only animals with bacteria in their lungs showed evidence of septicaemia (Fig. 2H).

The bacterial burden in the URT of animals challenged with SPN 10 days after viral infection remained relatively stable over time (Fig. 2C). Despite this finding, 3/9 animals were euthanised 72 hours after SPN challenge. Of the remaining six ferrets in this group, bacteria were recovered from the lungs of three animals at the end of the experiment (day 6 post-SPN) (Fig. 2F), but the number of lung lobes containing detectable SPN was less in animals with prior A(H3N2) compared to A(H1N1)pdm09 infection (2/5 lobes in 3/9 ferrets vs. 5/5 lobes in 5/6 ferrets, respectively). Septicaemia was evident in the same three animals with bacteria detected in their lungs and they were euthanised 72 hours post-SPN challenge (Fig. 2H).

For the first 4 days after SPN challenge, animals infected 16 days earlier with A(H3N2) showed bacterial loads in the URT comparable to those of control animals inoculated with SPN alone (Fig. 2D). After this time, bacterial titres in the URT were 10-fold higher compared to SPN alone controls and 2/6 animals required euthanasia. Bacteria were detected in one or more lung lobes of 5/6 animals in this group at the time of euthanasia (day 5 post-SPN challenge; Fig. 2F), however, despite this, no animals were septicaemic (Fig. 2H). Together, these data suggest that animals infected with the A(H3N2) virus were susceptible to SBI-induced disease for at least 16 days after viral infection, which is substantially longer than animals previously infected with A(H1N1)pdm09. These data suggest that the duration of susceptibility to subsequent challenge with SPN is longer following A(H3N2) compared to A(H1N1)pdm09 infection in the ferret model, at least for the virus strains used in this study.

## Discussion

Individuals with viral infections presenting concurrently with community-acquired pneumonia (CAP) occur at a frequency between 30% and 50% in both adult and paediatric populations (Voiriot et al. 2016, Cawcutt and Kalil 2017). A total of two-thirds of patients presenting with sepsis have viral coinfections and are misdiagnosed by clinicians (Ljungström et al. 2017, Burrell et al. 2018), most likely due to the difficulty in identifying symptoms. Insight regarding the duration of susceptibility to SBIs following influenza virus infections could improve diagnosis and lead to better clinical outcomes if the relationship between the two was better understood. In addition, a more aggressive treatment regime to minimise the effects of the primary influenza virus infection may also reduce the incidence and/or impact of SBIs. Observational studies conducted during the 2009 A(H1N1)pdm09 pandemic demonstrated that oseltamivir treatment within 48 hours of symptom onset improved survival in patients with severe influenza and may have reduced SBIs (Kumar 2011).

In this study, we utilised a model where influenza virus-infected ferrets were challenged with SPN at various times postinfection with either human A(H3N2) or A(H1N1)pdm09 viruses to investigate the duration of susceptibility to SPN-induced disease. Irrespective of the IAV subtype used, animals remained highly susceptible to SPN and experienced severe disease when challenged with SPN 5–16 days after influenza virus infection. Viral replication in the LRT was not required for the development of pneumonia and/or septicaemia. Furthermore, we found that animals infected with A(H3N2) virus remained susceptible to SPN infections for a longer period (at least 16 days) when compared to animals infected with A(H1N1)pdm09 virus, at least for the virus strains used in our studies.

Mortality data from pneumonia and influenza includes combined deaths from primary viral infections as well as secondary bacterial pneumonia due to the difficulty in accurately identifying the causative agent. Using pneumonia and influenza data from 26 influenza seasons (1970–1995) in the UK, A(H3N2) viruses were associated with higher rates of hospitalisations compared to A(H1N1) influenza viruses (Simonsen et al. 2000). Statistical modelling using viral surveillance and mortality data from 1976–1977 through to 1998–1999 from the United States has shown that more deaths and a higher rate of secondary pneumonia occurred when A(H3N2) viruses predominated circulated (Thompson et al. 2003). More recently in Australia, epidemiological studies showed that during the 2017 influenza season, A(H3N2) infections resulted in an unprecedented number of ICU administrations due to viral and bacterial pneumonia, and these were higher than those observed during the peak of the A(H1N1)pdm09 influenza pandemic (Burrell et al. 2018). Our findings that ferrets infected with A(H3N2) virus were more susceptible to subsequent SPN-induced disease for extended periods compared to A(H1N1)pdm09-infected animals could provide some insight regarding the elevated hospitalisations and deaths that occur when A(H3N2) viruses predominate in the influenza season. Clearly, studies using additional virus strains are required to consolidate our initial findings in ferrets, however, it is of interest to note that replication of A(H3N2) viruses in the lungs did not appear to be an absolute requirement for subsequent SPN-induced disease.

Differences in the duration of susceptibility of IAV-infected animals to subsequent SBI challenge highlight the potential contributing role of virus-specific virulence factors. The hemagglutinin (HA) surface glycoprotein can contribute to tropism for particular cell types in the ferret airways based on the type and linkage of sialic acid receptors that they express (Cheng et al. 2012), while the enzymatic activity of the viral neuraminidase (NA) cleaves sialic acid residues, including those expressed by glycoproteins and glycolipids in the mucus layers, enabling penetration of virus to the epithelium layer for subsequent replication (Zanin et al. 2016). Additionally, the NA strips sialic acid receptors from the lung epithelium, further exposing receptors for pneumococci (McCullers and Bartmess 2003). In the mouse model of SBI, the NA enzymic activity is directly proportional to the rate of bacterial adherence (Peltola et al. 2005) with higher enzymic activity resulting in enhanced bacterial adherence. In a direct comparison, N2 NA glycoproteins were shown to be more active than N1 (Peltola et al. 2005), which could partially explain the enhanced duration of susceptibility to SBIs seen in our study when using an A(H3N2) virus. Influenza-induced epithelial cell death also exposes more attachment sites for SPN adherence (Plotkowski et al. 1986), and direct binding of the virus to the bacterium can also facilitate adhesion to epithelial cells (Rowe et al. 2020). NA inhibitors only partially protected mice from bacterial-induced complications after influenza infection (McCullers and Bartmess 2003, McCullers 2004).

Other factors may also contribute to differences in the type and intensity of immune responses to H3N2 compared to H1N1 viruses such as virally triggered changes in the bacterial microbiome that occur before the SPN infection, which may differentially facilitate the success of SPN colonisation. In support of this possibility, a household transmission model of the influenza virus, susceptibility to A/H3N2 and IBVs was associated with the microbiome in the nose and throat of individuals (Tsang et al. 2020). The study postulated that different oligiotypes of bacteria could impact the host susceptibility to influenza viral infections, therefore, the microbiome can modulate your risk of infection (Tsang et al. 2020). The results from these studies could also be extended to complications associated with SBIs and is an area of interests for future studies. Additionally, this study highlights the need to investigate IBVs and SPN infection.

For animals infected with A(H3N2) prior to challenge with SPN 10 or 16 days later, the bacterial load in the URT was comparable to control animals challenged with SPN alone, however, they were still prone to increased disease severity in some animals. It is worth noting that the animals infected with A(H3N2) may remain susceptible to SBIs for longer than 16 days following influenza infection, and this should be explored in future studies. Additionally, the bacterial lung burden was lower in animals infected with A(H3N2) virus compared to A(H1N1)pdm09. Previous studies have shown that human A(H3N2) viruses more readily induced sinusitis and otitis media, whereas seasonal A(H1N1) viruses showed a tendency to induce pneumonia in ferrets (Peltola et al. 2006). Thus, it is possible in our model that prior infection with A(H3N2) was also associated with elevated bacterial loads in the middle ear, however this was not examined.

Furthermore, we have used only one SPN strain (19F strain), which is known to colonise the nasopharynx of children and can also cause invasive pneumococcal disease, however, > 97 SPN strains have been described (Petraitiene et al. 2015, Ganaie et al. 2020) and their colonisation abilities may differ. As discussed above, our studies also focussed on the use of a single A(H1N1)pdm09 and A(H3N2) strain for the majority of experiments described, and there is likely to be variation between different influenza strains in their ability colonise the lungs and to promote susceptibility to SBI.

In summary, we have used a ferret model based on SPN challenge at various times after IAV infection to investigate the duration of susceptibility to SBI-induced disease following infection with different IAV subtypes. Our findings that A(H3N2) infection was associated with a longer period of susceptibility to subsequent SPN-induced disease are consistent with epidemiological data indicating that A(H3N2) viruses have caused more severe infections in humans. Understanding the window of SBI susceptibility in the context of influenza infections is essential in reducing the burden of disease caused by these dual infections. Our findings also highlight the need for additional studies to determine if aggressive influenza antiviral drug treatment combinations could be used to reduce the period of susceptibility to SBIs and their more severe outcomes. Reducing the incidence and impact of SBI following influenza infections could have major public health benefits such as reducing hospitalisations and deaths during annual influenza epidemics and potential future pandemics.
